# Clinicopathological characteristics and fertility preserving treatment of atypical polypoid adenomyoma

**DOI:** 10.3389/fonc.2024.1386931

**Published:** 2024-05-28

**Authors:** Qian Wang, Weiwei Shan, Bingyi Yang, Yu Xue, Yaochen Lou, Xiaojun Chen

**Affiliations:** ^1^ Obstetrics and Gynecology Hospital, Fudan University, Shanghai, China; ^2^ Shanghai Key Laboratory of Female Reproductive Endocrine Related Diseases, Obstetrics and Gynecology Hospital of Fudan University, Shanghai, China

**Keywords:** atypical polypoid adenomyoma, fertility preservation, endometrial atypical hyperplasia, endometrioid endometrial carcinoma, follow-up

## Abstract

**Objective:**

Atypical polypoid adenomyoma (APA) is a rare benign tumor frequently diagnosed in young women that may coexist with or progress to atypical endometrial hyperplasia (EAH) or endometrioid endometrial carcinoma (EEC). This study aimed to investigate which subset of patients with APA are prone to concurrent or subsequent EAH or EEC, evaluate the necessity of progestin treatment in patients with APA only after achieving a complete response (CR) through hysteroscopic lesion resection, and assess the impact of concurrent APA on the fertility-preserving treatment of EAH or EEC.

**Methods:**

This retrospective single-center study analyzed 86 patients with APA treated at the Obstetrics and Gynecology Hospital of Fudan University between January 2010 and October 2021. Patients with EAH or EEC only who underwent fertility-preserving treatment during the same period were matched in a 2:1 ratio with patients with concurrent APA and EAH or EEC. The clinicopathological characteristics, treatments, and prognosis were analyzed.

**Results:**

The median patient age was 31 years (range 21–47 years). Among the 86 included patients, nine underwent total hysterectomy, 62 received conservative treatment, and the remaining 15 were lost to follow-up. A comparison of the 16 patients with APA only versus the 58 patients with APA and concurrent or subsequent EAH or EEC revealed that a homeostasis model assessment of insulin resistance (HOMA-IR) of > 2.2 (P = 0.047) and high-density lipoprotein (HDL) concentration of < 1.2 mmol/L (P = 0.028) were independent risk factors for EAH or EEC in patients with APA. Among the 17 patients with APA only who received conservative treatment and achieved a CR after hysteroscopic lesion resection, 13 received hormone treatment for a median duration of 6.3 months. The median follow-up time for these 17 patients was 49.0 months, during which no recurrence of APA was observed, but six patients developed endometrial hyperplastic diseases. Regarding the impact of concurrent APA on fertility-preserving treatment for EAH or EEC, the median time to achieve a CR was 24.0 weeks (95% confidence interval [CI]: 23.0–40.4) in the APA group and 26.0 weeks (95% CI: 24.3–32.3) in the non-APA group (P = 0.424). There were no significant differences between the two groups in the outcomes of fertility-preserving treatment.

**Conclusion:**

Patients with APA only may still develop endometrial hyperplastic diseases after complete resection of the lesion under hysteroscopy to achieve a CR, particularly those with a HOMA-IR of > 2.2 or HDL concentration of < 1.2 mmol/L. Concurrent APA did not affect the efficacy of fertility-preserving treatment in patients with EAH or EEC.

## 1 Introduction

Atypical polypoid adenomyoma (APA) is a rare, benign uterine disease characterized by polypoid lesions (1) composed of atypical, architecturally complex endometrial glands set within benign myomatous or fibromyomatous stroma (2). It is usually observed in premenopausal women with a mean age of 39 years (3–5), and its clinical presentation is mainly abnormal uterine bleeding (6). Although the pathogenesis of APA is unclear, some cases may arise secondary to prolonged estrogenic stimulation (7). APA is reported to have a high risk (approximately 8.8%) of developing into endometrial adenocarcinoma or endometrial atypical hyperplasia (EAH) (8).

As APA is a rare disease, several questions remain unanswered. First, APA carries a high risk of coexistence with or development of EAH or endometrioid endometrial carcinoma (EEC). However, the specific subset of patients with APA prone to concurrent or subsequent EAH or EEC remains unclear. Second, it is uncertain whether APA without concurrent endometrial hyperplastic lesions requires progestin treatment after hysteroscopic complete resection of the APA lesions. Some studies have demonstrated that progestin therapy after hysteroscopic resection prevents recurrence and the progression to endometrial cancer in patients with APA compared with untreated patients (9–12). In contrast, other studies report that progestin therapy is ineffective (13, 14). Third, the impact of concurrent APA on the outcomes of fertility-preserving treatment in patients with EAH and EEC has not been thoroughly explored.

To answer these questions, we conducted a single-center retrospective study. We analyzed the clinicopathological characteristics of patients with APA and analyzed the long-term outcomes of follow-up after achieving complete response (CR) in patients diagnosed with APA only who underwent conservative treatment. Furthermore, we explored the potential risk factors associated with the coexistence or subsequent occurrence of EAH or EEC in patients with APA and evaluated the impact of APA on fertility-preserving treatment outcomes in EAH and EEC.

## 2 Materials and methods

### 2.1 Study population

This study was approved by the Institutional Review Board (IRB, No. 2021-204) of our hospital. A total of 121 consecutive patients diagnosed with APA by dilation and curettage (D&C), hysteroscopy, or total hysterectomy at the Obstetrics and Gynecology Hospital of Fudan University between January 2010 and October 2021 were retrospectively screened for study eligibility. Forty-one patients were excluded owing to a lack of basic demographic data or initial clinical information. To meet the objectives of our study, we included four groups of patients in the analysis:

1. The ‘APA only’ group consisted of patients initially diagnosed with APA without concurrent endometrial hyperplastic lesions. If these patients underwent conservative treatment, they remained free from endometrial hyperplasia during follow-up.
2. The ‘APA with EAH or EEC’ group comprised patients who were initially diagnosed with APA and concurrent EAH or EEC, as well as those who developed EAH or EEC during the follow-up period.
3. The ‘fertility-preservation for APA with EAH/EEC’ group included patients initially diagnosed with APA concurrent with EAH or EEC who opted for fertility-preserving treatment.
4. The ‘fertility-preservation for EAH/EEC only’ group comprised patients with EAH or EEC without APA who underwent fertility-preserving treatment. These patients were matched in a 2:1 ratio with those in the ‘fertility-preservation for APA with EAH/EEC’ group treated during the same treatment period to assess the impact of APA on fertility-preserving treatment of EAH or EEC.

To be considered eligible for fertility-preserving treatment, patients had to be histologically confirmed of EAH or well-differentiated EEC (grade 1); have no signs of extrauterine involvement, myometrial, or cervical invasion; be younger than 45 years of age; have a strong willingness to preserve their fertility; have no contraindications to progestin treatment or pregnancy; not be pregnant; and have good compliance with treatment.

An elevated body mass index (BMI) was considered an independent risk factor for failure of fertility-preserving treatment (15–17). To ensure comparability, we used the BMI and treatment method as matching criteria. Patients from both fertility-preserving groups were paired if their BMI values differed by less than 2 and they received the same treatment. The patients received progestin-based treatment, including megestrol acetate (MA, 160 mg orally per day), MA combined with metformin (0.5 g orally three times per day), MA combined with the levonorgestrel intrauterine system (LNG-IUS), MA combined with the LNG-IUS and metformin, and gonadotropin-releasing hormone analog (GnRH-a) (hypodermic injection every 4 weeks) and letrozole (160 mg orally per day). The detailed information of the patients in the two groups is shown in **Table 1**; **Supplementary Tables S2**, **S3**.

**Table 1:** General characteristics of patients with EAH or EEC with or without APA who received fertility-preserving treatment.

variables	Total (N=93)	fertility -preservation for APA with EAH/EEC (n=31)	fertility -preservation for EAH/EEC only (n=62)	*P* value *
**Age, years**	30(26-42)	29(22-37)	30(26-42)	0.157
**BMI (kg/m^2^)**	22.13(16.38-33.13)	21.23(16.80-32.05)	22.30(16.38-33.13)	0.483
≥28	15(16.1)	5(16.1)	10(16.1)	1.000
<28	78(83.9)	26(83.9)	52(83.9)	/
**HOMA-IR**	2.00 (0.22-10.51)	1.96(0.22-4.48)	2.07(0.84-10.51)	0.071
>2.95	23(24.7)	6(19.4)	17(27.4)	0.395
≤2.95	70(75.3)	25(80.6)	45(72.6)	/
**Waist-hip ratio**	0.84(0.65-0.98)	0.84(0.71-0.97)	0.84(0.65-0.98)	0.990
**Metabolic syndrome (%)**	28(30.1)	10(32.3)	18(29.0)	0.749
**Hypertension (%)**	14(15.1)	4(12.9)	10(16.1)	0.918
**Diabetes (%)**	4(4.3)	0	4(6.5)	0.366
**CA125(IU/ml)**	19.20(6.84-91.79)	20.31(6.84-72.38)	17.82(7.37-91.79)	0.665
**Nulliparous**	82(88.2)	30(96.8)	52(83.9)	0.140
**Treatment options (%)**	/	/	/	0.958
MA ^a^	72(77.4)	24(77.4)	48(77.4)	
MA+MET ^b^	12(12.9)	4(12.9)	8(12.9)	
MA+LNG-IUS ^c^	7(7.5)	2(6.5)	5(8.1)	
Others	2(2.2)	1(3.2) ^d^	1(1.6) ^e^	

Data are shown as number (%) or median (range).

p-value: comparison between APA with EAH or EEC and EAH or EEC only group.

a. MA (160 mg orally taken per day).

b. MA (160 mg orally taken per day) +MET (0.5g orally taken three times per day).

c. MA (160 mg orally taken per day) +LNG-IUS.

d. GnRHa (hypodermic injection every four weeks) + Letrozole (160 mg orally taken per day).

e. MA (160mg qd) +MET (0.5g tid) +LNG-IUS.

APA, atypical polypoid adenomyoma; EAH, endometrial atypical hyperplasia; EEC, endometrioid endometrial carcinoma; BMI, body mass index; HOMA-IR, homeostasis model assessment-insulin resistance; CA-125, cancer antigen 125; MA, megestrol acetate; MET, metformin; LNG-IUS, levonorgestrel intrauterine system; GnRHa, gonadotropin-releasing hormone analog.

### 2.2 Diagnosis and assessment

The pathological diagnosis was confirmed by two experienced gynecological pathologists according to the World Health Organization pathological classification (2020). If their opinions differed, a panel discussion was held to reach a final diagnosis.

Demographic, clinical, and pathological data at the time of the first diagnosis and follow-up information were collected. The lesion location was determined by hysteroscopy, total hysterectomy, or ultrasonography. Lesion size was defined as the maximum diameter measured using ultrasonography or pathological examination. Fasting blood glucose (FBG), fasting insulin (FINS), blood lipid, and cancer antigen 125 (CA-125) levels were assessed. BMI and homeostasis model assessment of insulin resistance (HOMA-IR) index were calculated, and metabolic syndrome (MS) criteria were evaluated (17). Overweight or obesity is defined as a BMI (weight in kilograms divided by the square of height in meters) ≥ 25 kg/m^2^. HOMA-IR index (FBG [mmol/L] × FINS [μU/mL]/22.5) was used to evaluate insulin resistance status. MS is diagnosed when an individual has at least three of the following: abdominal obesity (waist circumference ≥ 80 cm), high blood pressure (systolic blood pressure ≥ 130 mmHg or diastolic blood pressure ≥ 85 mmHg), high FBG (glucose levels ≥ 5.6 mmol/L or diagnosed type 2 diabetes), high triglyceride (TG) levels (levels ≥ 1.7 mmol/L), and low high-density lipoprotein (HDL) cholesterol (levels < 1.3 mmol/L).

### 2.3 Treatment and follow-up

The included patients received conservative treatment or hysterectomy according to their individual conditions. The data were prospectively recorded during the treatment and follow-up periods.

For patients with APA only who underwent conservative treatment, CR was characterized by the successful hysteroscopic removal of the lesion, followed by confirmation of normality on ultrasound or histopathological examination. Subsequently, these patients received one of the following treatment regimens: high-dose progestin, oral contraceptive pills, or observation. Ultrasonography was performed every 3–6 months during the follow-up period, and an endometrial biopsy using the Pipelle curette, D&C, or hysteroscopy was performed if necessary. The recurrence rate of APA, rate of endometrial hyperplasia development, pregnancy rate, and live birth rate were analyzed.

For patients with EAH or EEC, regardless of the presence of concurrent APA, hysteroscopic evaluation and resection of the lesions were performed every 3–4 months during treatment. The evaluation of the treatment response was categorized as CR, partial response (PR), stable disease (SD), and progressive disease (PD). CR was defined as the absence of endometrial lesions on pathological examination. PR was characterized as the pathological improvement, which encompassed the regression of EAH into complex or simple hyperplasia, or the regression of EEC into atypical, complex, or simple hyperplasia. SD was defined as the persistence of lesions consistent with the original diagnosis. PD was delineated as the presence of either evidence of endometrial cancer in patients initially diagnosed with EAH or evidence of a higher pathological grade, myometrial invasion, or extrauterine metastasis in patients initially diagnosed with EEC. Ultrasonography and endometrial biopsy using Pipelle were performed at follow-up every 3–6 months. Enhanced pelvic magnetic resonance imaging was performed and serum CA-125 levels were measured as indicated. The median time to achieve CR, cumulative CR rate at 16 and 32 weeks of treatment, recurrence rate after CR, pregnancy rate, and live birth rate were analyzed. All patients were followed-up until January 31, 2022.

### 2.4 Statistical analysis

Categorical variables were compared using the chi-square test or Fisher’s exact test and are presented as frequencies and percentages. The intragroup differences for continuous variables were compared using the Student’s t-test or Mann-Whitney U-test, as appropriate. Univariate and multivariate logistic regression analyses were performed to explore the risk factors for patients with APA with EAH or EEC. Therapeutic duration was estimated using the Kaplan–Meier method and compared between groups using the log-rank test. Two-sided tests were considered statistically significant at p < 0.05. All statistical analyses were performed in SPSS (Version 26.0; IBM Corp, Armonk, NY, USA). 

## 3 Results

### 3.1 Patient characteristics

**Figure 1:**
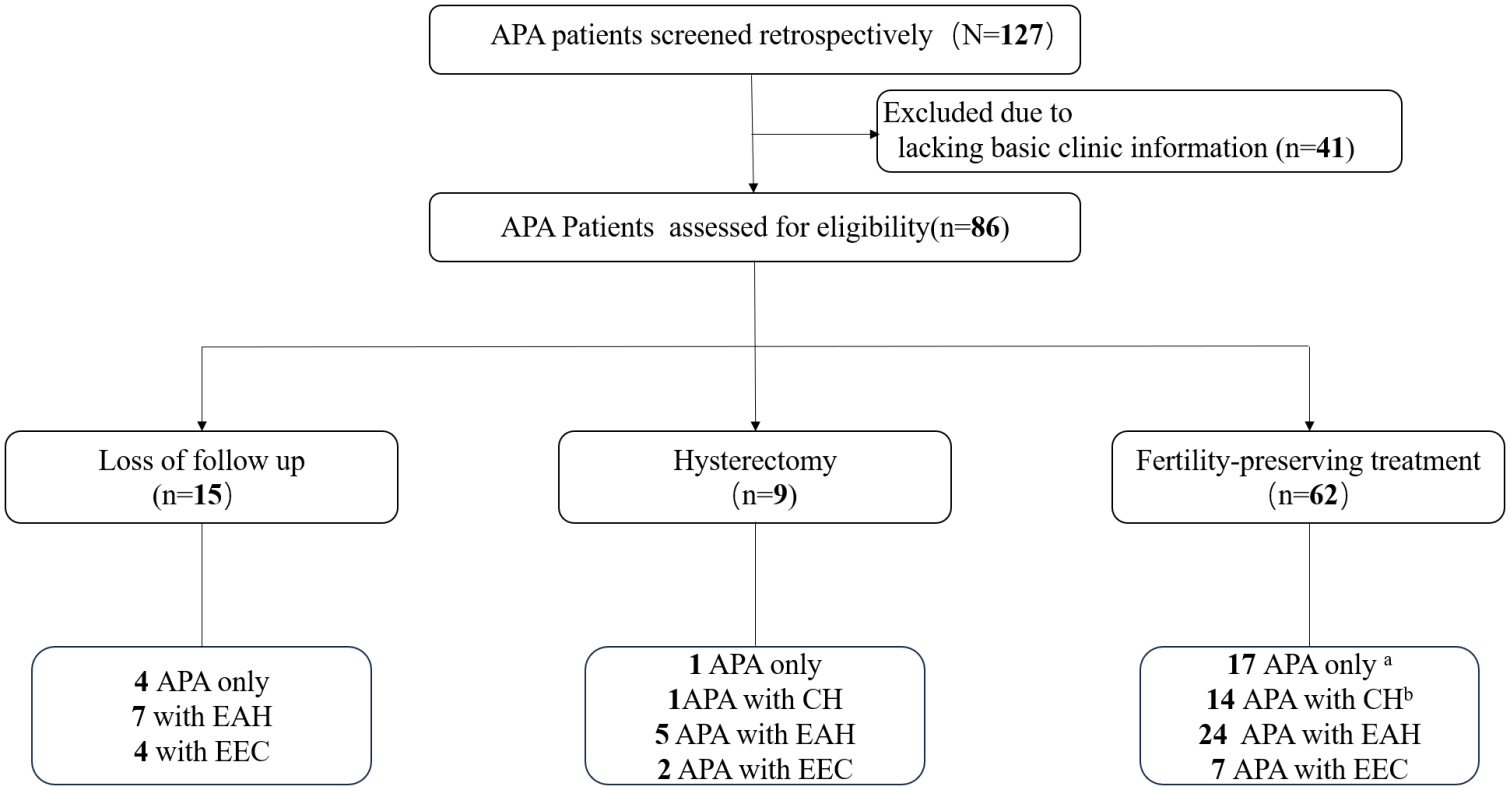
Flow diagram. Four patients developed EAH and two developed CH during follow-up after complete resection of APA. Four patients developed EAH and one patient developed EEC during follow-up. APA, atypical polypoid adenomyoma; EAH, endometrial atypical hyperplasia; EEC, endometrioid endometrial carcinoma; CH, complex hyperplasia.

A total of 86 patients with APA were included in this study (**Figure 1**). During the initial assessment, 22 patients (25.6%) were diagnosed with APA only, while 15 (17.4%) with concurrent complex hyperplasia (CH), 36 (41.9%) with concurrent EAH, and 13 (15.1%) with concurrent EEC. Among the 86 patients included, fifteen patients were lost to follow-up after the initial visit, and nine patients underwent total hysterectomy. Among the 62 patients who received fertility-preserving treatment, four (4/17, 23.5%) initially diagnosed with APA only developed EAH, and two developed CH during follow-up after hysteroscopic complete resection of the APA lesions. Similarly, four patients (4/14, 28.5%) initially diagnosed with APA with concurrent CH developed EAH, while one patient (1/14, 7.1%) developed EEC during follow-up. Consequently, nine patients were included in the APA with EAH or EEC group and two patients in the APA with CH group for basic characteristic analysis and investigation of risk factors associated with the coexistence or development of EAH or EEC in patients with APA.

The demographic and clinical characteristics of the patients are summarized in **Table 2**. The median age of the patients was 31 years (21-47 years), with 86% (74/86) being nulliparous. Abnormal uterine bleeding was observed in 65.1% (56/86) of the patients. APA lesions were mainly localized at the uterine fundus in 65.5% (55/84) of cases, at the lower uterine segment in 15.5% (13/84), at the cervix in 11.9% (10/84), and at both the uterine fundus and cervix in 7.1% (6/84) of the cases. The median lesion diameter was 2.1 cm (0.4-7.5 cm). Metabolic syndrome was found in 21.3% (16/75) of the patients. The median BMI of all patients was 22.42 kg/m^2^ (16.80-32.42 kg/m^2^). The median HOMA-IR was 1.98 (0.22-7.43). Median serum TG, total cholesterol (TC), low-density lipoprotein (LDL), HDL, and CA125 levels were within the normal range.

**Table 2:** General characteristics of patients with APA with or without EAH or EEC.

Variables	Total (N=86)	APA only (n=16)	APA with CH (n=12)	APA with EAH or EEC (n=58)	*P* value*
**Age, years**	31 (21-47)	34 (23-47)	29 (24-47)	30.5 (21-43)	0.063
**Nulliparous (%)**	74/86 (86.0)	13/16 (81.2)	10/12 (83.3)	51/58 (87.9)	0.680
**Symptom (%)**					0.296
Abnormal uterine bleeding	56/86 (65.1)	8/16(50.0)	9/12(75.0)	39/58 (67.2)	
Infertility	11/86(12.8)	2/16(12.5)	1/12 (8.3)	8/58 (13.8)	
Asymptomatic physical examination	13/86 (15.1)	5/16(31.3)	1/12 (8.3)	7/58 (12.1)	
Others	6/86 (7.0)	1/16(6.2)	1/12(8.3)	4/58 (6.9)	
**Location of APA (%)** †					0.610
Uterine fundus	55/84 (65.5)	11/16 (68.8)	10/12 (83.3)	34/56 (60.7)	
Lower uterine segment	13/84 (15.5)	1/16 (6.2)	1/12 (8.3)	11/56 (19.7)	
Cervix	10/84 (11.9)	2/16 (12.5)	1/12 (8.3)	7/56 (12.5)	
Uterine fundus and cervix	6/84 (7.1)	2/16 (12.5)	0	4/56 (7.1)	
**lesion size(cm)**†	2.10 (0.40-7.50)	2.35 (0.40-5.80)	2.00 (0.70-3.00)	2.25 (0.50-7.50)	0.834
**Metabolic syndrome (%)** †	16/75 (21.3)	2/12 (16.7)	1/11 (9.1)	13/52 (25.0)	0.715
**Waist hip ratio**†	0.83(0.71-0.97)	0.85 (0.76-0.92)	0.79 (0.71-0.87)	0.83 (0.71-0.97)	0.734
**BMI (kg/m^2^)**	22.42(16.80-32.42)	21.50(17.07-27.34)	20.78 (16.80-31.29)	23.23 (16.80-32.42)	0.133
≥25	28/86 (32.6)	4/16 (25.0)	2/12 (16.7)	22/58 (37.9)	0.337
<25	58/86 (67.4)	12/16 (75.0)	10/12 (83.3)	36/58 (62.1)	
**HOMA-IR**†	1.98(0.22-7.43)	1.58 (0.78-2.82)	1.79 (0.47-5.42)	2.07 (0.22-7.43)	0.028
**TG (mmol/L)** †	0.79 (0.45-5.11)	0.70 (0.51-3.58)	0.83 (0.48-3.20)	0.88 (0.45-5.11)	0.059
**TC (mmol/)** †	4.41 (2.87-6.47)	4.36 (2.87-6.06)	4.58 (3.40-6.47)	4.41(2.97-6.34)	0.667
**HDL (mmol/)** †	1.08 (0.56-2.24)	1.38 (0.76-2.24)	1.09 (0.85-1.72)	1.07 (0.56-1.97)	0.050
**LDL (mmol/)** †	2.80 (0.64-4.67)	2.83(1.46-3.95)	2.95(1.64-4.67)	2.71(0.64-4.24)	0.213
**CA125(IU/ml)** †	19.18 (5.49-104.00)	15.01 (5.49-70.67)	18.30 (7.95-31.80)	20.27 (6.84-104.00)	0.689

Data are shown as number (%) or median (range).

*P-value for comparison between patients with APA with EAH or EEC and APA-only.

†All variables were analyzed among the 86 patients, except for the location of APA, lesion size, waist-to-hip ratio, metabolic syndrome, HOMA-IR, TG, TC, HDL, LDL, and CA125. Data for two cases for the location of APA, 23 for lesion size, 20 for waist-to-hip ratio, 11 for metabolic syndrome, 10 for HOMA-IR, six for TG, seven for TC, six for HDL, six for LDL and eight for CA125 were missing.

BMI, body mass index; HOMA-IR, homeostasis model assessment insulin resistance; CA-125, cancer antigen 125; APA, atypical polypoid adenomyoma; EAH, endometrial atypical hyperplasia; EEC, endometrioid endometrial carcinoma; TG, triglyceride; TC, total cholesterol; HDL, high-density lipoprotein; LDL, low-density lipoprotein.

### 3.2 Risk factors for developing EAH or EEC in patients with APA

The demographic and clinical characteristics of the patients with APA only and those with EAH or EEC are compared in **Table 2**. Patients with EAH or EEC had a higher HOMA-IR (P = 0.028) than those with APA only. There were no statistically significant differences in the distribution of other characteristics, including age, symptoms, location of APA, lesion size, BMI, waist-hip ratio, serum lipids, and CA125 levels.

**Figure 2:**
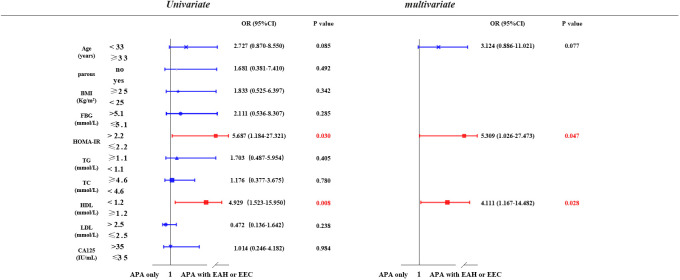
Possible risk factors related to APA with EAH or EEC. Univariate and multivariate logistic regression models were used to identify risk factors associated with the development of EAH or EEC in patients with APA. APA, atypical polypoid adenomyoma; EAH, endometrial atypical hyperplasia; EEC, endometrioid endometrial carcinoma; BMI, body mass index; FBG, fasting Blood glucose; HOMA-IR, homeostasis model assessment insulin resistance; TG, triglyceride; TC, total cholesterol; HDL, high-density lipoprotein; LDL, low-density lipoprotein; CA-125, cancer antigen 125.

We investigated the potential risk factors associated with the coexistence or development of EAH or EEC in patients with APA (**Figure 2**). Through univariate analysis, we observed a positive correlation between elevated HOMA-IR (> 2.2) (P = 0.030) and lower serum HDL (< 1.2 mmol/L) (P = 0.008) with the presence of EAH or EEC in patients with APA. This association persisted in the multivariate analysis after adjusting for age, HOMA-IR, and HDL. Specifically, patients with APA with a HOMA-IR of > 2.2 were 5.31 times (95% confidence interval [CI] = 1.03-27.47) more likely to develop EAH or EEC compared to those with a HOMA-IR ≤ 2.2 (P = 0.047). Similarly, patients with APA with a HDL concentration < 1.2 mmol/L faced a 4.11-fold (95% CI= 1.17-14.48) increased risk of developing EAH or EEC compared to those with a HDL concentration≥ 1.2 mmol/L.

### 3.3 Long-term follow-up outcome of patients with APA only after CR

Seventeen patients initially diagnosed with APA only received conservative treatment and achieved CR after complete resection of the lesion under hysteroscopy. As shown in **Table 3**, four patients were followed-up under observation; six patients received high-dose progestin therapy, which included MA 160 mg per day orally in five patients and LNG-IUS placed in one patient, while seven patients received cyclic treatment with Diane-35. Detailed treatment durations and follow-up lengths are provided in **Supplementary Table S1**. The median treatment duration was 4.5 months in the high-dose progestin group and 12.6 months in the Diane-35 group. The median follow-up time for the all 17 patients was 49.0 months (30.1-55.5 months), during which none experienced recurrence of APA. However, six patients (6/17, 35.3%) developed endometrial hyperplasia, including two cases of CH and four cases of EAH. The median duration from CR to the diagnosis of CH or EAH was 32.0 months (19.8-57.0). Among these six patients, three who received high-dose progestin therapy developed EAH, with a median treatment duration of approximately three months of MA; two patients who received cyclic Diane-35 therapy developed CH, and one developed EAH. Twelve patients had fertility requirements, with pregnancy rates of 3/4 (75%) in the observation group, 3/3 (100%) in the high-dose progestin group, and 3/5 (60%) in the Diane-35 group. All pregnant women delivered live births, except for one patient in the observation group who experienced a miscarriage.

**Table 3:** The follow-up results of patients with APA only after complete resection of lesion.

	Total (n=17)	observation	high-dose progestin	Diane-35
		(n=4)	(n=6)	(n=7)
**Time of drug treatment, Mo**	6.3 (3.1-15.7)	/	4.5 (3.0-26.0)	12.6 (3.1-18.2)
**Follow-up time**†**, Mo**	49.0 (30.1-55.5)	18.0 (7.1-50.5)	64.1 (41.2-79.6)	36.2 (16.5-64.1)
Develop into endometrial hyperplastic disease (%)				
CH	2(11.8)	0	0	2 (28.6)
EAH	4(23.5)	0	3 (50.0)	1 (14.3)
total	6 (35.3)	0	3(50.0)	3 (42.9)
**Time of developing into endometrial hyperplastic diseases, Mo**	32.0 (19.8-57.0)	/	36.9 (24.0-75.7)	27.2 (15.6-38.4)
**Pregnant rate (%)**	9/12 (75.0)	3/3 (100.0)	3/4 (75.0)	3/5 (60.0)
**live birth rate (%)**	8/9 (88.9)	2/3 (66.7)	3/3 (100.0)	3/3 (100.0)

†Time was presented as median and 95% confidence interval.

APA, atypical polypoid adenomyoma; EAH, endometrial atypical hyperplasia; CH, complex hyperplasia; Mo, months.

### 3.4 Impact of APA on the fertility-preserving treatment in patients with EAH or EEC

A total of 24 patients with EAH and 7 patients with EEC diagnosed with APA (‘fertility -preservation for APA with EAH/EEC’ group) who received fertility-preserving treatment were analyzed. Controls comprised 48 patients with EAH and 14 patients with EEC without APA (‘fertility-preservation for EAH/EEC only’ group). The general characteristics of patients are shown in **Table 1**. No significant differences were observed between the two groups regarding age, BMI, waist-to-hip ratio, CA125 level, complications (IR, MS, diabetes, and hypertension), parity, or fertility-preserving treatment.

**Figure 3:**
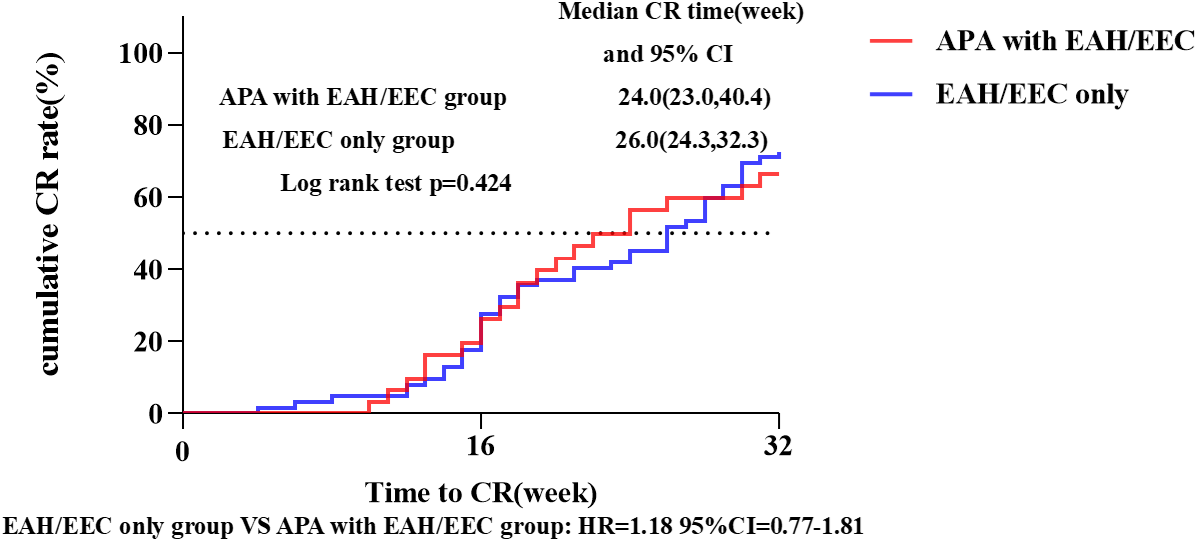
CR rate and median CR time in patients with EAH or EEC with or without APA. Kaplan-Meier survival curves for cumulative CR rate in patients who received fertility-preserving treatment. CI, confidence interval; CR, complete response; APA, atypical polypoid adenomyoma; EAH, endometrial atypical hyperplasia; EEC, endometrioid endometrial carcinoma; HR, hazard ratio.

The median follow-up duration for all patients was 42.4 months (39.8-47.4 months). In the ‘fertility -preservation for APA with EAH/EEC’ group, the median time to achieve CR was 24.0 weeks (95% CI 23.0-40.4), compared to 26.0 weeks (95%CI 24.3-32.3) in the ‘fertility-preservation for EAH/EEC only’ group, with no significant difference observed between the two groups (log-rank test, P = 0.424; hazard ratio = 1.18, 95% CI 0.77-1.81) (**Figure 3**). There were no significant differences in the cumulative CR rate at 16 weeks (22.6% *vs*. 27.4%, P = 0.615) and 32 weeks (64.5% *vs*. 70.5%, P = 0.560), recurrence rate (30.0% *vs*. 26.7%, P = 0.739), pregnancy rate (47.8% *vs*. 58.5%, P = 0.409), and live birth rate (72.7% *vs*. 62.5%, P = 0.550) between the two groups (**Table 4**).

**Table 4:** Fertility-preserving treatment outcomes of patients with EAH or EEC with or without APA.

	Total (N=93)	fertility -preservation for APA with EAH/EEC (n=31)	fertility -preservation for EAH/EEC only (n=62)	P value
**16-week cumulative CR rate (%)**	24 (25.8)	7 (22.6)	17 (27.4)	0.615
**32-week cumulative CR rate (%)**	63(68.5)	20(64.5)	43(70.5)	0.560
**Recurrence rate (%)**	25/90(27.8)	9/30(30.0)	16/60(26.7)	0.739
**Follow-up time**†**, Mo**	42.4(39.8-47.4)	38.9(35.6-51.5)	43.2(39.4-47.8)	0.993
**Pregnancy rate (%)**	35/64(54.7)	11/23(47.8)	24/41(58.5)	0.409
**Live birth rate (%)**	23/35(65.7)	8/11(72.7)	15/24(62.5)	0.550

† Follow-up time were presented as median time and 95% confidence interval.

CR, complete response, APA, atypical polypoid adenomyoma; EAH, endometrial atypical hyperplasia; EEC, endometrioid endometrial carcinoma; Mo, months.

Similar results were obtained when assessing the impact of APA on fertility-preserving treatment in patients with EAH or EEC (**Supplementary Tables S2**–**S5**, **Supplementary Figure S1**).

## 4 Discussion

This single-center retrospective study demonstrated that an elevated HOMA-IR (> 2.2) and lower HDL concentration (< 1.2 mmol/L) are independent risk factors for EAH or EEC in patients with APA. Furthermore, patients with APA only may still develop endometrial hyperplastic diseases during follow-up after achieving a CR and receiving short-term hormone therapy. However, concurrent APA did not appear to have an impact on the outcomes of fertility-preserving treatment in patients with EAH or EEC.

In our study, all 86 patients diagnosed with APA were premenopausal, with a median age of 31 (21-47) years. Abnormal uterine bleeding was the most common symptom, while a minority of patients were identified with infertility (12.8%) or diagnosed incidentally during physical examination (15.1%). The median lesion diameter of APA was 2.1(0.4-7.5) cm, and it was commonly observed at the uterine fundus, lower uterine segment, and cervix, with the uterine fundus being the predominant site (65.5%). These characteristics were consistent with those reported in previous literatures (3–5).

Our study revealed that 56.9% of patients with APA were diagnosed with concurrent EAH or EEC. Heatley et al. (8) reported the prevalence of APA (8.8%) concurrent with endometrial hyperplasia and adenocarcinoma. The lower prevalence of APA coexisting with EAH or EEC in their previous study compared with our study may be attributable to our study’s retrospective nature and the exclusion of patients lacking clinical and demographic information. Nonetheless, our findings strongly suggest that patients with APA are predisposed to developing endometrial hyperplastic diseases, particularly EAH or EEC. Some studies have reported that APA shares several risk factors with endometrial cancer, such as obesity, diabetes, hypertension, and high estrogen concentrations, which can lead to the concomitant presence of both conditions (18, 19). However, the reasons for the progression of APA to cancer are still unclear and require further study. In our research, we found that a HOMA-IR of > 2.2 and HDL concentration of < 1.2 mmol/L were significantly associated with the occurrence of EAH or EEC in patients with APA. Therefore, patients with APA only with a HOMA-IR of > 2.2 and HDL concentration of < 1.2 mmol/L require rigorous monitoring.

As APA is a benign disease, complete lesion resection through hysteroscopy is accepted as the first-line treatment for patients with APA without concurrent endometrial hyperplastic lesions (20). However, it is unclear whether hormone treatment after achieving a CR is helpful in preventing the recurrence of APA or the development of endometrial hyperplastic diseases. In our study, we did not observe the recurrence of APA after a median follow-up of 49 months in the 17 patients who achieved a CR, regardless of the use of hormone treatment. However, we observed that a total of six individuals developed endometrial hyperplastic diseases: three patients who received high-dose progestin treatment developed EAH, two patients who underwent Diane-35 cyclic therapy developed CH, and one patient treated with Diane-35 developed EAH. A previous retrospective study of 55 patients who underwent hysteroscopic transcervical resection to comprehensively remove lesions reported a significantly lower postoperative recurrence rate (9.1% *vs*. 36.4%) and malignant transformation rate (3.0% *vs*. 18.2%) in the treated group versus the untreated group (12), providing evidence to support the use of progestogen in the treatment of APA. Additionally, two studies by Nomura et al. (11, 21) reported that hormonal therapy with medroxyprogesterone acetate is associated with a reduced recurrence rate in patients with APA. In contrast, a multicenter observational retrospective cohort study of 24 patients reported that conservative treatment for APA achieved long-term success in 92.9% of patients who received hysteroscopy alone and 72.3% of patients who underwent hysteroscopy plus progestin (14), indicating that hysteroscopy is the better treatment approach. Furthermore, a systematic review reported no significant differences between hysteroscopy alone and hysteroscopy with hormone maintenance in terms of the rate of cancer progression (10.8% *vs*. 5.1%) and recurrence rate (29.8% *vs*. 17.9%) (6). Therefore, the role of postoperative adjuvant hormone therapy for APA remains controversial. Although our results seem to suggest that short-term hormone therapy may not prevent APA, the limited number of cases in our study is insufficient to draw a definitive conclusion. Thus, we need to accumulate more cases to confirm our conclusions.

Although concurrent APA is common in patients with EAH or EEC, the impact of APA on the outcomes of conservative treatment in these patients is unknown. Our data showed that patients in the ‘fertility-preservation for APA with EAH/EEC’ group had similar rates of CR at 16 weeks and 32weeks, recurrence, pregnancy, and live birth compared with those in the ‘fertility-preservation for EAH/EEC only’ group. In our study, the median time to achieve a CR was 24.0 weeks (95% CI 23.0–40.4) and the cumulative CR rate at 32 weeks was 64.5% in the ‘fertility-preservation for APA with EAH/EEC’ group, which is consistent with the findings of previous studies on fertility-preserving treatment for patients with EAH or EEC (22–25). These data indicate that concurrent APA does not affect fertility-preserving treatment outcomes in patients with EAH or EEC. For this subset of patients, the treatment should be aimed at treating EAH or EEC only.

The present study investigated a relatively large number of patients with APA with concurrent EAH or EEC who received fertility-preserving treatment. We assessed the impact of concurrent APA on the efficacy of fertility-preserving treatment in these patients. Additionally, we analyzed the long-term outcomes of patients diagnosed with APA who underwent hysteroscopic lesion resection with or without subsequent hormone therapy. Our study had several limitations. First, its single-center retrospective nature and small sample size limited the power of the results. Second, the patients were recruited between 2010 and 2021, and some data were missing.

## 5 Conclusion

Our preliminary data showed that patients diagnosed with APA only may develop EAH or EEC even after complete lesion resection, particularly those with a HOMA-IR of > 2.2 or HDL concentration of < 1.2 mmol/L. Concurrent APA did not affect fertility-sparing treatment outcomes in patients with EAH or EEC. Further studies with larger sample sizes are required to validate our findings.

## Data availability statement

The original contributions presented in the study are included in the article/**Supplementary Material**. Further inquiries can be directed to the corresponding author.

## Ethics statement

The studies involving humans were approved by Institutional Review Board in Obstetrics and Gynecology Hospital of Fudan University. The studies were conducted in accordance with the local legislation and institutional requirements. The participants provided their written informed consent to participate in this study. Written informed consent was obtained from the individual(s) for the publication of any potentially identifiable images or data included in this article.

## Author contributions

QW: Data curation, Formal analysis, Investigation, Writing – original draft, Writing – review & editing. WS: Conceptualization, Data curation, Writing – review & editing. BY: Methodology, Writing – review & editing. YX: Methodology, Software, Writing – review & editing. YL: Formal analysis, Software, Writing – review & editing. XC: Conceptualization, Supervision, Writing – review & editing.
