# Supplementary material for: Clinicopathological characteristics and fertility preserving treatment of atypical polypoid adenomyoma
Source: Front Oncol. 2024 May 28;14:1386931. doi: 10.3389/fonc.2024.1386931 (PMC11165147; doi:10.3389/fonc.2024.1386931)
Supplement: Supplementary file 1 [file DataSheet_1.docx]

Supplementary Material

# Supplementary Figures and Tables

## Supplementary Figure


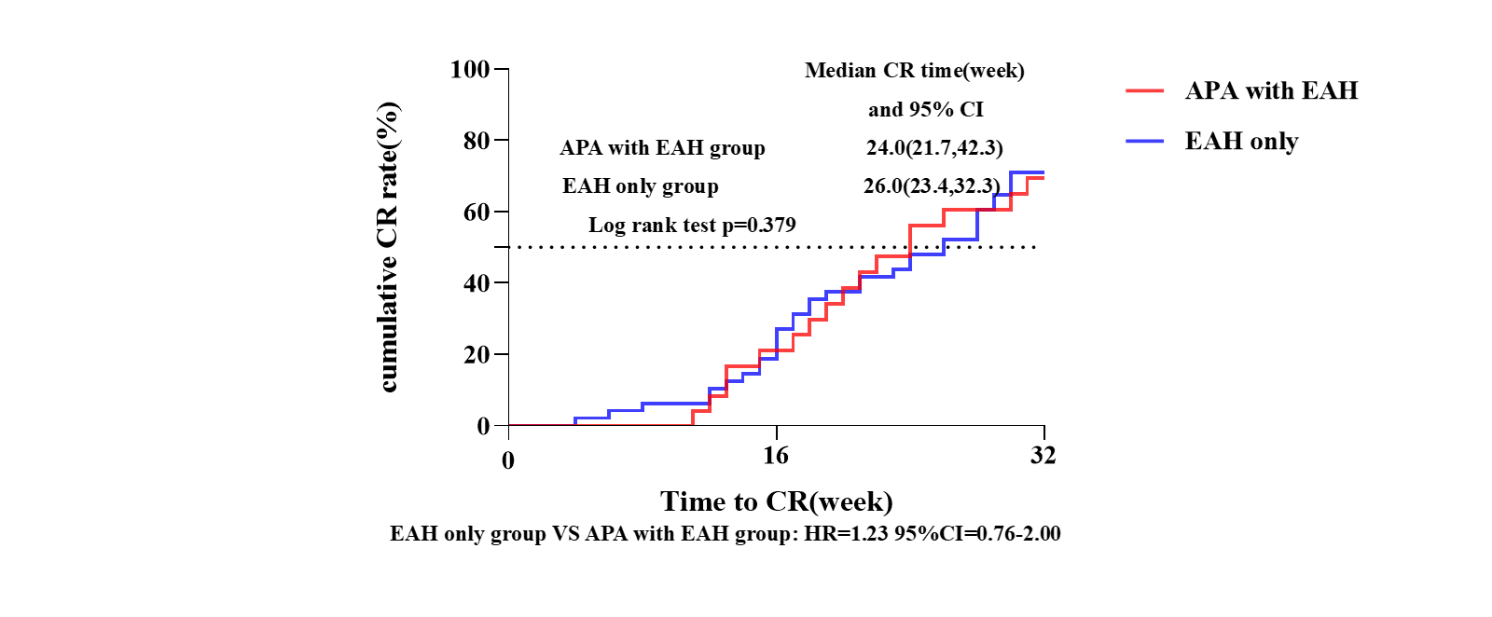


A

**
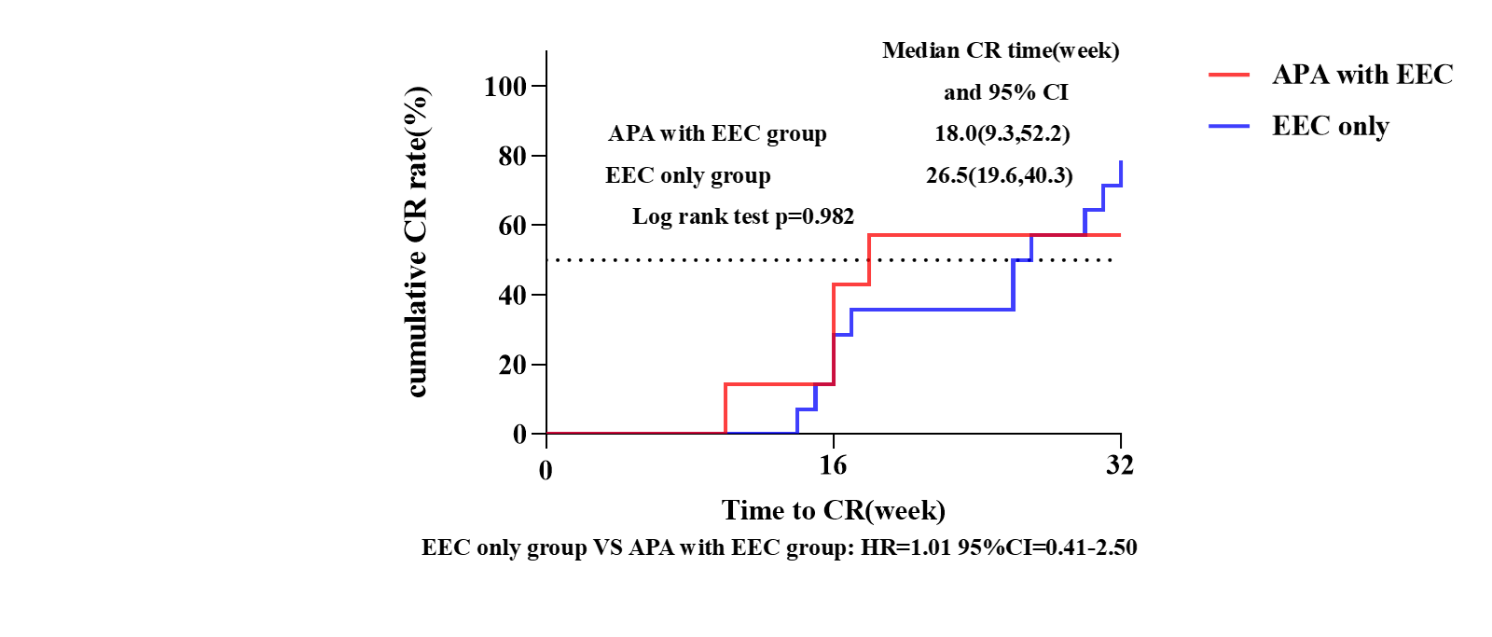
**

B

**Supplementary Figure S1.** CR rate and median CR time in patients with EAH (A) and EEC (B) with or without APA.

Kaplan-Meier survival curves for cumulative CR rate in patients who received treatment.

CI, confidence interval; CR, complete response; APA, atypical polypoid adenomyoma; EAH, endometrial atypical hyperplasia; EEC, endometrioid endometrial carcinoma; HR, hazard ratio.

## Supplementary Tables

**Table S1.** List of medication and follow-up of patients with APA only after complete resection of lesion.

| **number** | **method** | **length of medication (Mo)** | **follow up time (Mo)** | **Develop into endometrial hyperplasic disease** | **Time of developing into endometrial hyperplasic diseases (Mo)** |
| --- | --- | --- | --- | --- | --- |
| 1 | observation |  | 21.7 |  |  |
| 2 | observation |  | 7.5 |  |  |
| 3 | observation |  | 50.5 |  |  |
| 4 | observation |  | 14.2 |  |  |
| 5 | MA | 6.3 | 83.3 |  |  |
| 6 | MA | 5.1 | 33.5 |  |  |
| 7 | MA | 3.0 | 67.7 | EAH | 24.0 |
| 8 | MA | 3.0 | 60.4 | EAH | 36.9 |
| 9 | MA | 3.9 | 75.8 | EAH | 75.7 |
| 10 | LNG-IUS | 48.9 | 48.9 |  |  |
| 11 | Diane-35 | 12.6 | 66.7 | CH | 15.6 |
| 12 | Diane-35 | 2.0 | 51.1 | CH | 27.2 |
| 13 | Diane-35 | 16.5 | 16.5 |  |  |
| 14 | Diane-35 | 3.1 | 17.7 |  |  |
| 15 | Diane-35 | 36.2 | 36.2 |  |  |
| 16 | Diane-35 | 18.2 | 64.1 | EAH | 38.4 |
| 17 | Diane-35 | 7.2 | 11.7 |  |  |

APA, atypical polypoid adenomyoma; EAH, endometrial atypical hyperplasia; CH, complex hyperplasia; MA, megestrol acetate; LNG-IUS, levonorgestrel intrauterine system; Mo, months

**Table S2.** General characteristics of patients with EAH with or without APA who received fertility-preserving treatment.

| **variables** | **Total** | **fertility -preservation for APA with EAH（n=24）** | **fertility -preservation for EAH only**  **（n=48）** | ***P* value *** |
| --- | --- | --- | --- | --- |
| **Age, years** | 30(22-38) | 29(22-37) | 30(26-38) | 0.442 |
| **BMI (kg/m^2^)** | 21.67(16.38-33.1) | 20.61(16.80-32.05) | 22.09(16.38-33.13) | 0.519 |
| ≥28 | 12(16.7) | 4(16.7) | 8(16.7) | 1.000 |
| <28 | 60(83.3) | 20(83.3) | 40(83.3) |  |
| **HOMA-IR** | 1.98(0.22-5.84) | 1.60(0.22-3.54) | 2.11(0.84-5.84) | 0.051 |
| ＞2.95 | 17(23.6) | 4(16.7) | 13(27.1) | 0.327 |
| ≤2.95 | 55(76.4) | 20(83.3) | 35(72.9) |  |
| **Waist-hip ratio** | 0.84(0.65-0.97) | 0.84(0.71-0.97) | 0.84(0.65-0.97) | 0.995 |
| **Metabolic syndrome (%)** | 19(26.4) | 6(25.0) | 13(27.1) | 0.850 |
| **Hypertension (%)** | 12(16.7) | 3(12.5) | 9(18.8) | 0.737 |
| **Diabetes (%)** | 4(5.6) | 0 | 4(8.3) | 0.363 |
| **CA125(IU/ml）** | 19.75(6.84-91.79) | 19.10(6.84-72.38) | 20.30(7.37-91.79) | 0.884 |
| **Nulliparous (%)** | 61(84.7) | 23(95.8) | 38(79.2) | 0.132 |
| **Treatment options (%)** | 72(100) | / | / | 1.000 |
| MA | 57(79.1) | 19(79.1) | 38(79.1) |  |
| MA+MET | 9(12.5) | 3(12.5) | 6(12.5) |  |
| MA+LNG-IUS | 3(4.2) | 1(4.2) | 2(4.2) |  |
| Others | 3(4.2) | 1(4.2) | 2(4.2) |  |

Data are shown as number (%) or median (range); p-value: comparison between the APA with EAH and EAH only groups.

APA, atypical polypoid adenomyoma; EAH, endometrial atypical hyperplasia; BMI, body mass index; HOMA-IR, homeostasis model assessment-insulin resistance; CA-125, cancer antigen 125; MA, megestrol acetate; MET, metformin; LNG-IUS, levonorgestrel intrauterine system.

**Table S3.** General characteristics of patients with EEC with or without APA who received fertility-preserving treatment.

| **variables** | **Total （N=21)** | **fertility -preservation for APA with EEC （n=7）** | **fertility -preservation for EAH only**  **（n=14）** | ***P* value *** |
| --- | --- | --- | --- | --- |
| **Age, years** | 30.4±4.2 | 29.0±3.6 | 31.1±4.5 | 0.285 |
| **BMI (kg/m^2^)** | 22.59±3.69 | 22.25±4.11 | 22.76±3.61 | 0.773 |
| ≥28 | 3(14.3) | 1(14.3) | 2(14.3) | 1.000 |
| <28 | 18(85.7) | 6(85.7) | 12(85.7) |  |
| **HOMA-IR** | 1.92(0.88-10.51) | 2.21(1.83-4.48) | 1.78(0.88-10.51) | 0.094 |
| >2.95 | 6(28.6) | 2(28.6) | 4(28.6) | 1.000 |
| ≤2.95 | 15(71.4) | 5(71.4) | 10(71.4) |  |
| **Waist-hip ratio** | 0.84(0.72-0.98) | 0.84(0.73-0.88) | 0.81(0.72-0.98) | 0.913 |
| **Metabolic syndrome (%)** | 9(42.9) | 4(57.1) | 5(35.7) | 0.397 |
| **Hypertension (%)** | 2(9.5) | 1(14.3) | 1(7.1) | 1.000 |
| **Diabetes (%)** | 0 | 0 | 0 | / |
| **CA125(IU/ml）** | 17.81(7.27-79.40) | 20.31(7.27-57.90) | 17.15(10.90-79.40) | 0.689 |
| **Nulliparous (%)** | 21(100) | 7(100) | 14(100) | / |
| **Treatment options (%)** | / | / | / | 1.000 |
| MA | 15(71.4) | 5(71.4) | 10(71.4) |  |
| MA+MET | 3(14.3) | 1(14.3) | 2(14.3) |  |
| MA+LNG-IUS | 3(14.3) | 1(14.3) | 2(14.3) |  |

Data are shown as number (%) or median (range); p-value: comparison between the APA with EEC and EEC only groups.

APA, atypical polypoid adenomyoma; EEC, endometrioid endometrial carcinoma; BMI, body mass index; HOMA-IR, homeostasis model assessment-insulin resistance; CA-125, cancer antigen 125; MA, megestrol acetate; MET, metformin; LNG-IUS, levonorgestrel intrauterine system.

**Table S4.** The outcomes of fertility-preserving treatment in patients with APA with EAH and EAH only.

|  | **Total**  **（N=72)** | **APA with EAH**  **（n=24）** | **EAH only**  **（n=48）** | **P value** |
| --- | --- | --- | --- | --- |
| **16-week cumulative CR rate** **(%)** | 18(25.4) | 5(21.7) | 13(27.1) | 0.628 |
| **32-week cumulative CR rate (%)** | 49(70.0) | 16(69.6) | 33(70.2) | 0.956 |
| **Recurrence rate (%)** | 17/69(24.6) | 6/23(26.1) | 11/46(23.9) | 0.843 |
| **Time to recur**†**, Mo** | 24.5(14.0-33.7) | 15.6(7.1-47.7) | 24.7(12.1-51.0) | 0.525 |
| **Follow-up time**†**, Mo** | 43.3(38.3-47.3) | 49.2(28.8-57.2) | 43.15(38.3-46.4) | 0.773 |
| **Pregnancy rate (%)** | 26/45(57.8) | 8/17(47.1) | 18/28(64.3) | 0.257 |
| **Live birth rate (%)** | 18/26(69.2) | 7/8(87.5) | 11/18(61.1) | 0.156 |

†Time to recur and follow-up time are presented as median time and 95% confidence interval.

CR, complete response, APA, atypical polypoid adenomyoma; EAH, endometrial atypical hyperplasia; Mo, months

**Table S5.** The outcomes of fertility-preserving treatment in patients with APA with EEC and EEC only.

|  | **Total**  **（N=21)** | **APA with EEC**  **（n=7）** | **EEC only**  **（n=14）** | **P value** |
| --- | --- | --- | --- | --- |
| **16-week cumulative CR rate (%)** | 6(28.6) | 2(28.6) | 4(28.6) | 1.000 |
| **32-week cumulative CR rate (%)** | 14(66.7) | 4(57.1) | 10(71.4) | 0.638 |
| **Recurrence rate (%)** | 8(38.1) | 3(42.9) | 5(35.7) | 1.000 |
| **Time to recur**†**, Mo** | 11.2(3.7-45.7) | 10.0(3.8-13.8) | 12.3(3.7-45.7) | 0.399 |
| **Follow-up time**†**, Mo** | 34.5(25.9-51.3) | 25.8(14.1-58.8) | 42.3(26.5-65.1) | 0.104 |
| **Pregnancy rate (%)** | 9/19(47.4) | 3/6(50.0) | 6/13(46.2) | 0.876 |
| **Live birth rate (%)** | 5/9(55.6) | 1/3(33.3) | 4/6(66.7) | 0.341 |

†Time to recur, and follow-up time were presented as median time and 95% confidence interval.CR, complete response, APA, atypical polypoid adenomyoma; EEC, endometrioid endometrial carcinoma; Mo, months
